# Assessing uncertainties from physical parameters and modelling choices in an atmospheric large eddy simulation model

**DOI:** 10.1098/rsta.2020.0073

**Published:** 2021-05-17

**Authors:** Fredrik Jansson, Wouter Edeling, Jisk Attema, Daan Crommelin

**Affiliations:** ^1^ Centrum Wiskunde & Informatica, Amsterdam, Netherlands; ^2^ Delft University of Technology, Delft, Netherlands; ^3^ Netherlands eScience Center, Amsterdam, Netherlands; ^4^ Korteweg-de Vries Institute, University of Amsterdam, Amsterdam, Netherlands

**Keywords:** atmospheric modelling, large eddy simulation, uncertainty quantification

## Abstract

In this study, we investigate uncertainties in a large eddy simulation of the atmosphere, employing modern uncertainty quantification methods that have hardly been used yet in this context. When analysing the uncertainty of model results, one can distinguish between uncertainty related to physical parameters whose values are not exactly known, and uncertainty related to modelling choices such as the selection of numerical discretization methods, of the spatial domain size and resolution, and the use of different model formulations. While the former kind is commonly studied e.g. with forward uncertainty propagation, we explore the use of such techniques to also assess the latter kind. From a climate modelling perspective, uncertainties in the convective response and cloud formation are of particular interest, since these affect the cloud-climate feedback, one of the dominant sources of uncertainty in current climate models. Therefore we analyse the DALES model in the RICO case, a well-studied convection benchmark. We use the VECMA toolkit for uncertainty propagation, assessing uncertainties stemming from physical parameters as well as from modelling choices. We find substantial uncertainties due to small random initial state perturbations, and that the choice of advection scheme is the most influential of the modelling choices we assessed.

This article is part of the theme issue ‘Reliability and reproducibility in computational science: implementing verification, validation and uncertainty quantification *in silico*’.

## 1. Introduction

Atmospheric moist convection and cloud dynamics are physical processes that have small spatial scales yet they are important for the behaviour of atmosphere and climate. They are crucial for the transport of moisture through the atmosphere and thus for the global hydrological cycle, and despite their small scales they have an impact on the large-scale atmospheric circulation. Moreover, the behaviour of convection and clouds plays a significant role in climate change projections because of the cloud-climate feedback [1,2].

Modelling and simulation of convection and cloud processes is complex because of their range of spatio-temporal scales, the complexity of the physics involved (including fluid dynamics, thermodynamics and cloud particle microphysics) and the nonlinearity of their interactions. In global atmosphere models, these processes cannot be explicitly resolved; instead their effect is represented through parameterizations. The uncertainties stemming from parameterizations are known to be a major source of uncertainty in climate change projections [3,4]. In local models, with limited spatial domain but sufficiently high resolution to resolve convection explicitly, uncertainties arise because of the complexities of modelling and simulation mentioned above. Assessing these uncertainties is important, for example because simulation results from local models are used intensively for designing, studying and validating parameterizations.

In this study, we explore and assess the uncertainties of a convection-resolving large eddy simulation (LES) model of the atmosphere, making use of modern tools for uncertainty quantification (UQ) [5,6]. We focus our attention on the Dutch atmospheric LES (DALES) model [7], a model used in many previous studies of atmospheric convection [8–12], and apply the model to the RICO benchmark case [11]. For convection to be well resolved in DALES, its horizontal grid resolution must be set at 200 m or smaller. The horizontal domain size varies between studies, with typical values of 10–50 km.

The numerical output from the DALES model has multiple sources of uncertainty. We discern several types among these sources. We believe that these types, discussed below, are encountered in many other applications and problems involving computational models.

First of all, the model has *physical input parameters* whose values are not precisely known. Examples of these in the DALES model are cloud droplet concentration, temperature at the lower boundary of the simulation domain (earth or sea surface) and surface roughness length. Uncertainties in such physical input parameters are the most common in UQ studies. However, further uncertainties stem from what we call *model choices*: choices that must be made for starting a simulation, for example between different schemes (or modules) for cloud microphysics that are used in the overall DALES model, or between different numerical schemes available for advection. A third, related type of uncertainty is due to *numerical settings*, such as domain size and spatial resolution. Also, the DALES model can be used with an iterative Poisson solver whose tolerance must be set. The quantitative effect that these numerical settings have on model results is often unclear, so they can be regarded as sources of uncertainty.

We note that for some of these choices/settings it is clear from a theoretical perspective what the best choice is, however that information is not always helpful. For example, higher spatial resolution is in principle superior to lower resolution, as it gives more accurate results. But it also comes at a higher computational cost. What the optimal compromise is between cost and accuracy can be hard to determine, and the impact of a change in accuracy on the simulation output difficult to quantify.

Finally, the simulation results depend on a *random seed* used to initialize the random number generator. After picking an initial state for the DALES model, a small amount of noise is added to this state (e.g. to break unwanted symmetries). The noise is produced by the random number generator. This is a form of initial state uncertainty that warrants ensemble simulations, with different ensemble members initialized with different random seeds. Operational weather forecasts already routinely use ensembles. In research settings ensembles are not yet routinely used for LES models, although this is changing with more operational LES set-ups such as LASSO [13], the KNMI Parameterization Testbed [14] and the Ruisdael Observatory [15].

Some of the main tools for UQ are intended for uncertainty propagation, where the aim is to characterize the probability distribution of the simulation output given the distribution of uncertain physical input parameters. In this study, we demonstrate that these tools can also be very useful for assessing uncertainties in output that are due to model choices and numerical settings. These choices and settings do not have probability distributions associated with them.

The study of uncertainties in weather and climate modelling is not a new topic, e.g. [16–18]. However, many of these studies focus on global models and their predictions. The uncertainties in local process models for e.g. convection and clouds have been much less explored. Furthermore, the use of modern UQ tools such as stochastic collocation in this context is very new; the only previous study that we are aware of in this area is [19]. We note that the focus in that study is different from ours; for example, uncertainties due to model choices and numerical settings are not considered there.

We make use of a newly developed software toolkit VECMAtk [20] for assessing uncertainties in the DALES model. This toolkit is set up in a way that facilitates the use of high-performance computing resources. It can handle numerical models such as DALES in a black-box fashion and does not require extensive coding or model code adaptation. The Python code for the experiments in this article, connecting EasyVVUQ and DALES, is available online under an open source license. This code together with the results presented here can serve as a starting point for further UQ analysis of atmospheric models and in particular LESs.

In the following section, we introduce the DALES model, the UQ methods used, and the experiment set-up and discuss the model parameters chosen for variation. Section 3. shows the results of the UQ experiments, while §4. concludes the paper with a discussion of the influence of the varied parameters on the model results. In particular, we note that the sea surface temperature and the choice of the advection scheme have strong influences. We also show that some model results, especially the rain amount, vary between runs started with slightly different initial states, and recommend using ensembles rather than single model runs in order to measure the sensitivity of the results to such fluctuations.

## 2. Model and implementation

The DALES model simulates atmospheric processes such as turbulence, convection and clouds in a local domain. The model keeps track of the atmospheric state with three-dimensional grids of state variables, the most important of which describe the air velocity, temperature and humidity [7]. The extent of the domain is generally 10–50 km, with periodic boundary conditions applied in the horizontal directions.

In order to run a simulation with DALES, three forms of input are needed: a file with physical parameters and model settings, the state of the atmosphere at the start of the simulation period, and optionally, large-scale tendencies, i.e. information on how the conditions change over the simulation time due to the influence of weather phenomena on scales larger than the simulation domain.

The initial state of the model is usually provided in the form of vertical profiles of the model state variables. The model settings, the initial state and the large-scale tendencies together define a case. The initial profiles and large-scale tendencies can be obtained from other models on a larger scale [14,21–23] or be constructed from observations, e.g. during a field campaign, as in our case which is described below.

### (a) The RICO case

As a basis for the UQ experiments, we use the RICO case [11], a well-studied case for LES modelling, with cumulus clouds and rain over the ocean, based on a field observation campaign [24]. We chose the RICO case, since it is computationally affordable while simultaneously being a case of scientific interest.

We run the case as described in the reference, with a resolution of 100 m in the horizontal direction and 40 m in the vertical, and with a grid extent of 128 × 128 × 126 cells. We use the default DALES surface scheme based on Monin–Obukhov similarity theory instead of the simplified set-up in the case definition. This way, we can work with the official DALES version, and can include the surface roughness length parameter *z*_0_ in the study. With these settings one run of the case simulating 24 h lasts around 3 h, when run with 16 MPI tasks on a single computer node. It is thus computationally feasible to perform hundreds of runs, which is required for a comprehensive UQ analysis with several uncertain parameters at once.

### (b) Forward uncertainty propagation with EasyVVUQ

We deal with the forward propagation of uncertainties through DALES, and the subsequent approximation of sensitivity to input uncertainties. With forward uncertainty propagation, we assess the uncertainty in the DALES model output (denoted *q* below) given the uncertainties in the model input parameters (denoted *ξ* below). Related to the issue of uncertainty propagation is the question of how much of the total model output uncertainty can be ascribed to individual model inputs, i.e. the sensitivity of the model output to different inputs.

For a formal definition of forward uncertainty propagation, consider a multivariate random variable $\xi \in R^{d}$, to which *d* independent probability distribution functions (pdfs) are ascribed, such that the joint pdf is given by

$$p(\xi )=\prod_{i=1}^{d}p(\xi_{i}).$$

Then, the purpose of uncertainty propagation is to assess the effect of p(ξ) on the output q(ξ) of the computational model. Specifically, we are interested in the first two statistical moments, given by

$$E[q]=\int_{\Omega }q(\xi )\cdot p(\xi )\;d\xi \;\mathrm{and}\;V\mathrm{ar}[q]=\int_{\Omega }{\left(q(\xi )-E[q]\right)}^{2}\cdot p(\xi )\;d\xi .$$

Here, Ω is the support of p(ξ). In addition to the statistical moments, uncertainty propagation can also aim to approximate the pdf of the output, given by *p*(*q*).

We employ the EasyVVUQ toolkit [25], which is a Python3 library for forward uncertainty propagation. It contains different sampling techniques to achieve this, and can be easily coupled to various plugins to run the code samples on HPC resources (we use Fabsim3 [26] for this purpose, for smaller runs on a local machine GNU Parallel can also be used [27]). In the EasyVVUQ toolkit, we use the stochastic collocation (SC) sampler, which is described next.

### (c) Stochastic collocation

The SC method creates a polynomial approximation of a quantity of interest (QoI) *q* in the stochastic space $\xi \in R^{d}$ via the following expansion:

2.1 $$q(\xi )\approx \tilde{q}(\xi )=\sum_{j=1}^{N_{p}}q(\xi_{j})L^{j}(\xi ).$$

Here, $q(\xi_{j})$ is the code output evaluated at a specific point $\xi_{j}$ in the stochastic domain. These code samples are interpolated to an arbitrary point ξ by the Lagrange interpolation polynomials L(ξ). In the case of more than one uncertain parameter (*d* > 1), *L* is built as a tensor product of one-dimensional Lagrange polynomials. We can therefore expand (2.1) as

2.2 $$\begin{array}{rl} q(\xi )\approx \tilde{q}(\xi_{1},\ldots ,\xi_{d}) & =\sum_{j_{1}=1}^{m_{1}}\cdots \sum_{j_{d}=1}^{m_{d}}q(\xi_{1}^{j_{1}},\ldots ,\xi_{d}^{j_{d}})L_{1}^{j_{1}}⊗\cdots ⊗L_{d}^{j_{d}}(\xi_{1},\ldots ,\xi_{d}),\\ \mathrm{where}\;L_{i}^{j_{k}}(\xi_{i}) & =\prod_{\begin{array}{c} i_{k}=1\\ i_{k}\neq j_{k} \end{array}}^{m_{i}}\frac{\xi_{i}-\xi_{i}^{i_{k}}}{\xi_{i}^{j_{k}}-\xi_{i}^{i_{k}}}. \end{array}$$

Note that the tensor product construction yields an exponential increase in the number of code evaluations with the number of dimensions, i.e. *N*_*p*_ = *m*^*d*^, where we have assumed an equal number of points in each dimension (*m*_*i*_ = *m*). This is known as the curse of dimensionality. That said, for a moderate number of variables, the SC method can display exponential convergence, thereby outperforming standard Monte Carlo sampling [28].

The SC method can be used to compute the first two statistical moments of q(ξ), and the interpolation approximation $\tilde{q}$ of (2.1) can serve as a cheap surrogate model for the code output, once all samples $q(\xi_{j})$ are computed. In addition, the Sobol sensitivity indices (described next) can be approximated as a post-processing step. The SC method is a mature means of UQ, and for more information we refer to [28,29].

### (d) Sobol indices

Sensitivity measures compute the sensitivity of a function *q*(**ξ**) (in our case the output of the DALES model), with respect to its inputs $\xi \in R^{d}$. Local sensitivity methods concern themselves with the sensitivity of the code output close to some fixed reference point **ξ**_0_ in the input domain, and are therefore not reliable far away from this point. Global sensitivity measures are meant to address this shortcoming. Sobol indices [30] are well-known variance-based sensitivity measures which assign independent probability density functions *p*(*ξ*_*j*_) over the relevant domain of each input *ξ*_*j*_, making it a global method. Sobol indices are derived from the analysis of variance (ANOVA) decomposition of *q*(**ξ**). This decomposes *q* into a sum of basis functions of increasing input dimension, which in long forms reads as

2.3 $$\begin{array}{cc} q(\xi ) & =q_{\emptyset }\\ & +q_{1}(\xi_{1})+\cdots +q_{d}(\xi_{d})\\ & +q_{12}(\xi_{1},\xi_{2})+q_{13}(\xi_{1},\xi_{3})+\cdots +q_{d-1\;d}(\xi_{d-1},\xi_{d})\\ & +\cdots \\ & +q_{1,\ldots ,d}(\xi_{1},\ldots ,\xi_{d}). \end{array}$$

A more concise notation is

2.4 $$q(\xi )=\sum_{u\in F}q_{u},$$

where *u* is a multi-index and F is the power set of U:={1,2,…,d}. Each basis function *q*_*u*_ captures the effect on the code output, obtained by varying the subset of parameters indexed by *u*. The Sobol indices *S*_*u*_ are the normalized variances of these basis functions, i.e.

2.5 $$S_{u}:=\frac{D_{u}}{D}.$$

Here, *D*_*u*_ is the variance of *q*_*u*_ and *D* is the total variance, $D:=V\mathrm{ar}[q]=\sum_{u\in U}D_{u}$ [30]. Similar to the given interpretation of the basis functions, each *D*_*u*_ measures the contribution of a given subset of parameters to the total variance, which makes (2.5) a sensitivity measure. For instance, *S*_1_ is the fraction of the variance obtained by varying *ξ*_1_ by itself. Higher-order interaction effects are also computed, such that *S*_12_ is the amount of variance attributed by simultaneously varying *ξ*_1_ and *ξ*_2_. Note that since all *D*_*u*_ are positive, the sum of all *S*_*u*_ equals one.

The authors of [31] developed a method to approximate the *S*_*u*_ by replacing the code output q(ξ) with the SC surrogate $\tilde{q}(\xi )$ (2.1), which is implemented in EasyVVUQ. We refer to [31] for the details on the procedure, and to [30] for a general mathematical description of the Sobol indices.

### (e) Uncertainty quantification set-up

In choosing output QoIs to analyse, the UQ methods based on polynomial interpolation are expected to work best on quantities that vary smoothly with the input parameters. We wish to avoid quantities that fluctuate strongly due to the model’s chaotic behaviour, and therefore select quantities that are averaged over space and time.

Of the quantities measured in the RICO study [11], we choose the following. The cloud cover *C* measures the fraction of the horizontal model area covered by clouds. The liquid water path LWP and rain water path RWP measure the mass of cloud droplets and rain, respectively, per unit surface area in the model. The cloud base height *z*_*b*_ gives the height of the lowest occurrence of cloud liquid water, while the inversion height *z*_*i*_ measures the height of the largest vertical temperature gradient. Further quantities are the surface precipitation rate *P*_srf_ which gives the rate of rainfall at the surface, and the surface fluxes of moisture and heat, *w*_*q*_ and *w*_*θ*_. The quantities are averaged over the model domain and over the last 4 h of the simulation., i.e. from hour 20 to 24. Additionally, we measure the required computing time *τ* (wall-clock time).

In order to keep the number of model runs manageable, we divide the parameters in three groups of related quantities. These groups and parameters are shown in table 1 together with their values in the RICO case and the ranges in which they are varied in this study. The parameter groups are briefly described below; for a full description of the parameters and how they affect the simulation, see [7].

**Table 1.** Overview of the model parameters studied, grouped by experiment. The table shows the default values in the RICO case, and the range in which they are varied. The parameters are assumed to have a uniform distribution over the range. The random seed is included as a parameter in every study. The surface roughness length in DALES is given by separate parameters for momentum and heat. When *z*_0_ is varied, we here set them equal for simplicity. For more details of the parameters, see the DALES model description [7].

parameter	symbol	default value	range
*physical parameters*			
cloud droplet concentration	*N*_*c*_	70 cm^−3^	[50, 100]
sea surface temperature	*θ*_*s*_	298.5 K	[298, 299]
surface roughness length	*z*_0_	1.6 × 10^−4^ m (mom.)	[1, 2] ×10^−4^
		3.2 × 10^−5^ m (heat)	
random seed	seed		
*model choices*			
microphysics scheme	microphys.	SB	KK00 (Khairoutdinov & Kogan)
			or SB (Seifert & Beheng)
advection scheme	adv.	2nd	2nd or 5th order
rain advection scheme	rain adv.	kappa	2nd or 5th order or kappa
random seed	seed		
*iterative Poisson solver*			
Poisson solver tolerance	*ϵ* = 10^−*d*^	N/A	[2, 13]
random seed	seed		

#### (i) Uncertain physical parameters

One of the traditional applications of UQ is estimating means and distributions of output QoIs from the model, when the model input parameters are uncertain and have given distributions. In our case, we select the following parameters: the cloud droplet concentration *N*_*c*_, which affects the formation of rain droplets, and is know to be different over land and over the ocean; the sea surface temperature *θ*_*s*_ which affects evaporation and convective processes; and the surface roughness length *z*_0_ which describes the surface structure, and accounts for friction between the air and the surface. The experiment is set up with a uniform and constant sea surface temperature, and for this reason, *θ*_*s*_ is considered an input parameter to the experiment. The parameter ranges are taken from the RICO case, as roughly the ranges in which the parameters varied during the campaign. In the case of *z*_0_, we use an estimated range for an ocean surface.

#### (ii) Random seed

The RICO case is initialized with specified vertical profiles for the atmospheric variables (the main ones describe wind velocity, temperature and humidity), in other words the initial model state is uniform in the horizontal direction. In order to break the symmetry of the system and allow convection to form, a small amount of random noise is added to the initial model fields (the amplitude is about 10^−3^ times the field average). This noise is generated with a pseudo-random number generator, which is initialized with a value called a random seed. For different seed values, the noise fields are different, which makes the simulation follow a different trajectory.

The turbulent atmosphere is a chaotic system, and as such any perturbation of the initial state or the parameters of the model can be expected to change the model trajectory, and have a large impact on the final state of the simulation. This is mitigated by choosing QoIs that are averaged over both space and time, nevertheless it is desirable to have a way of measuring the effect of the model’s chaotic behaviour on each QoI. For this reason, for every point in parameter space sampled, we here use a small ensemble consisting of 4–6 model runs, started with the same parameters but different random seeds.

In practice, we construct this ensemble by including the random seed as a discrete parameter to be varied in every experiment. Using the random seed as a parameter has two issues. First, the QoIs cannot be assumed to vary smoothly with the random seed, since the seed is discrete and also since even numerically adjacent seeds by design are supposed to give completely different output from the random number generator. Second, the QoI variations due to the model’s chaotic nature can be expected to be as large between runs which differ only in the seed value as between runs that differ in parameter values.

This means that even though Sobol indices are computed for the random seed parameter, their interpretation is less clear as they do not capture all of the effects of chaotic behaviour on the QoIs. What this method does provide is that, in the cases when the QoI uncertainty is related to one of the proper model parameters, the Sobol indices show this with a high value for the decisive parameter and a low value for the random seed.

When the chaotic variations dominate (as often turns out to be the case with rain quantities below) the significance of the first-order Sobol indices is less clear, they generally tend to be small thus not signifying any particular parameter as responsible for the uncertainty.

#### (iii) Model choices

The DALES model has several settings for choosing numerical schemes or alternative model formulations. These settings are chosen with model switches, which takes one value from a given set of options. They generally do not have a probability distribution, since they are simply chosen by the modeller, perhaps using experience of which model formulation is appropriate or works well in a given case.

However, the UQ framework can easily be used to measure the impact of these choices on the model output, and for this purpose we map switch settings to values from a discrete uniform distribution. For the RICO case, we vary the model advection scheme (two options), the advection scheme for rain quantities (three options) and the cloud microphysics scheme (the model for how cloud droplets merge and form rain drops, two options).

#### (iv) Numerical settings

Many models have parameters controlling the accuracy in various ways, such as the spatial resolution, the size of the model domain and the time step length. The impacts of LES domain size [9] and resolution [32] have been investigated previously. For the DALES model, we use UQ to measure the impact of another numerical parameter, the stopping tolerance for an iterative Poisson equation solver.

To find the pressure at every time step, a Poisson equation needs to be solved; see eqn (42) of the DALES model description paper [7,32,33]. There are many different algorithms that can be used, with the optimal method depending on the boundary conditions, and periodicity and size of the domain. DALES implements two different classes of algorithms (solvers): an exact solver, and an iterative solver.

The exact solver uses the periodicity of the domain to do a Fourier decomposition in the two horizontal directions. The resulting set of equations is only coupled in the vertical, and can be solved using e.g. Gaussian elimination. Although the algorithm is exact, the solution is an approximation because of the discretization of the derivatives and the Fourier transforms. It is computationally very efficient, but does not scale well on large distributed memory systems where the communication overhead of the Fourier transform becomes the main bottleneck.

Iterative solvers are not exact, but find a solution by continuously improving on a first guess. The iteration halts when a criterion is met, typically when the absolute difference between consecutive iterations is smaller than a set tolerance. An iterative solver is computationally not as efficient as the exact solver, often needing many iterations before convergence is reached. However, with a suitably chosen first guess the number of iterations can be kept low. An iterative solver also has a different communication pattern: instead of the many all-to-all communication calls needed for the Fourier decomposition, it requires mainly communication between neighbouring grid cells. For the PALM LES model [34], which also has an iterative solver as an option, the iterative solver is reported to be faster than their exact Fourier-based solver for grids larger than 2000^3^ cells. Finally, the iterative solvers are more flexible than the exact solver, offering the opportunity for further model development. They can be made to work with for instance non-periodic domains and complex orography.

For DALES, we use the iterative solvers provided by the Hypre library [35,36]. Hypre implements many algorithms with a single interface, and is conceptually compatible with DALES: it has a similar grid decomposition and memory layout, with a well-documented Fortran interface. We use the parallel semicoarsening multigrid solver (PFMG) on a structured grid, with two iterations of Red/Black Gauss–Seidel pre- and post-relaxation [37,38]. As first guess for the pressure, we reuse the pressure of the previous time step. Choosing the tolerance is a trade-off between computational speed and accuracy. Here, the iterative method is part of a larger model, and it is not obvious how the Poisson solver tolerance affects the model output and its accuracy, or how to choose it optimally.

## 3. Results

We quantify uncertainties in the DALES model by measuring how it responds to variation of input parameters. In this section, we show the results of our UQ experiments on the DALES model; how the various quantities of interest respond to changes in the parameters varied.

For each group of input parameters in table 1, we perform an experiment where the parameters in the group are varied together. The parameter groups are physical parameters, model choices and numerical settings. We let EasyVVUQ use the SC sampler to determine which points to sample in the parameter space. The points in parameter space form an *n*-dimensional grid where *n* is the number of parameters in the group. Choosing the number of points along each dimension is a balance between obtaining a good coverage of the parameter space and keeping the total number of model runs manageable; in this study, we use between two and seven points per dimension (two or three points when the parameter is discrete and we choose from a limited set of options, four to seven when sampling continuous values, depending on the total number of samples in the experiment).

For each experiment, we show a scatter plot where each QoI is plotted against every varied parameter. Each such plot presents all the model runs in the experiment, in order to show the influence of one particular parameter (on the horizontal axis) compared with the influence of all the other parameters (shown as the vertical scatter of the points). A small random perturbation is added in the horizontal direction, to give a better impression of the point cloud. These plots show whether any single parameter is decisive for a given QoI. A clear example is seen in the case of the Poisson solver tolerance in §3c where the *d* parameter is responsible for the variation of the wall-clock time QoI.

Also shown are tables with the standard deviation for each parameter over all the samples, and the Sobol indices from the UQ analysis, which show how a large fraction of the total variability in each QoI can be attributed to uncertainty in each parameter. The Sobol indices range from 0 to 1, with 0 indicating no dependence on the parameter, and 1 indicating that the parameter is responsible for all of the variation of the QoI in the experiment. We show all the first-order Sobol indices which measure the direct influence of each parameter in the tables.

We see that the rain-related quantities *P*_srf_ and RWP generally have the largest variation. Rain generation is sensitive to cloud organization, which may explain why the rain quantities show the strongest variation.

### (a) Physical parameters

**Figure 1.**
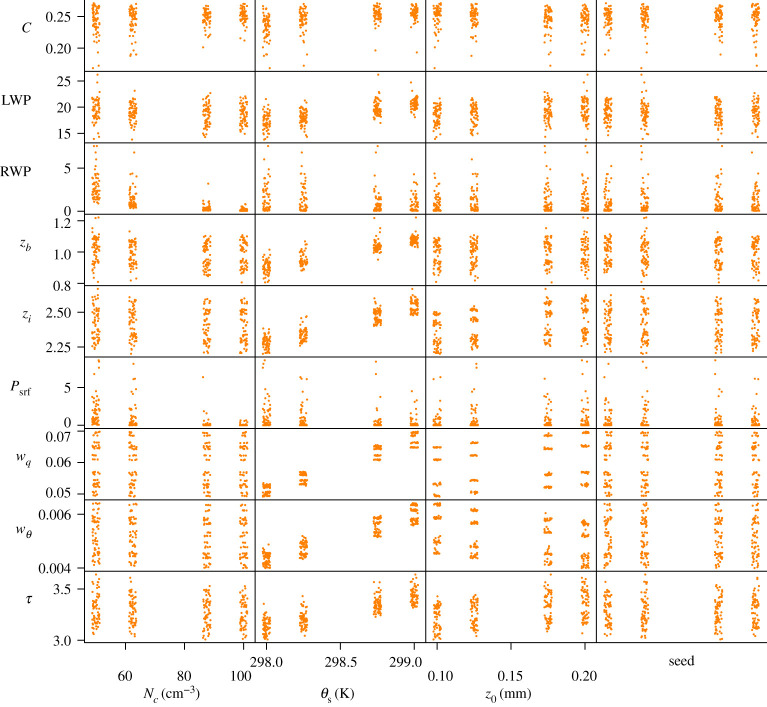
Influence of varying physical parameters (horizontal) on model output quantities (vertical). The varied parameters are the cloud droplet concentration *N*_*c*_, sea surface temperature *θ*_*s*_, surface roughness length *z*_0_ and the random seed. (Online version in colour.)

The effects of varying the physical parameters are shown in figure 1 and table 2. Both from the Sobol indices in the table and from the plots, it is seen that the sea surface temperature *θ*_*s*_ is the parameter responsible for most of the uncertainty. The surface fluxes *w*_*q*_ and $w_{\theta }$ for heat and moisture are seen to depend mostly on the sea surface temperature *θ*_*s*_, and to a smaller extent on the surface roughness length *z*_0_. The cloud droplet concentration *N*_*c*_ is seen to affect precipitation quantities: the surface precipitation rate *P*_srf_ and rain water path RWP. DALES keeps track of the total cloud water content of each grid cell, and uses the *N*_*c*_ parameter to calculate a cloud droplet size. Increasing *N*_*c*_ gives smaller cloud droplets, which in turn results in less rain being generated. The rain output quantities RWP and *P*_srf_ show large standard deviations while in particular for *P*_srf_ all first-order Sobol indices are small, which we attribute to variations between the different runs due to the model’s chaotic nature.

**Table 2.** Statistics (mean and standard deviation, std) and Sobol indices for varying physical parameters: the cloud droplet concentration *N*_*c*_, sea surface temperature *θ*_*s*_, surface roughness length *z*_0_ and the random seed. The italics quantities are discussed in the text.

QoI	mean	s.d. (%)	*N*_*c*_	*θ*_*s*_	*z*_0_	seed
*C*	0.248	4.8	0.030	0.151	0.043	0.000
LWP	18.5 g m^−2^	7.8	0.093	*0.415*	0.027	0.015
RWP	0.831 g m^−2^	*129.0*	*0.444*	0.051	0.005	0.022
*z*_*b*_	0.986 km	5.8	0.067	*0.735*	0.004	0.007
*z*_*i*_	2.4 km	3.5	0.005	*0.858*	0.094	0.001
*P*_srf_	0.459 W m^−2^	*254.8*	*0.141*	0.093	0.020	0.029
*w*_*q*_	0.0593 g kg^−1^ m s^−1^	8.1	0.000	*0.944*	*0.055*	0.000
*w*_*θ*_	0.00509 K m s^−1^	10.0	0.003	*0.874*	*0.118*	0.000
*τ*	3.27 h	3.4	0.018	0.672	0.141	0.006

### (b) Model choices

**Figure 2.**
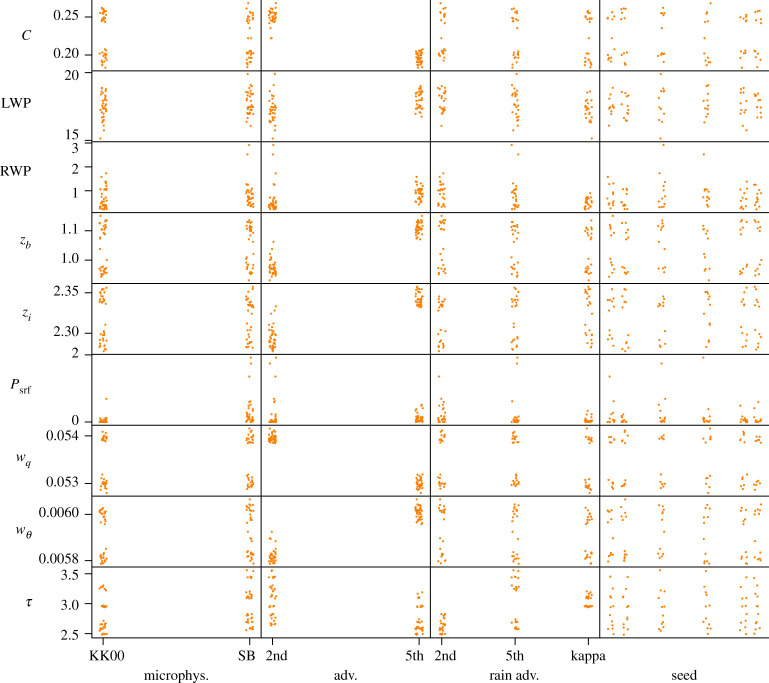
Influence of varying model choices (horizontal) on model output quantities (vertical). The varied parameters are the microphysics scheme, the advection schemes and the random seed. (Online version in colour.)

The effects of model choices are shown in figure 2 and table 3. Here, only the choice of advection scheme (adv.) is seen to have a strong effect. In particular the cloud fraction *C*, with a standard deviation of 9% over all the samples, depends strongly on the choice of advection scheme. The rain output quantities RWP and *P*_srf_ again show large standard deviations but small Sobol indices, which we attribute to variations between the different runs.

**Table 3.** Statistics and Sobol indices for varying model choices: the microphysics model, advection schemes and the random seed. The italics quantities are discussed in the text.

QoI	mean	s.d. (%)	microphys.	adv.	rain adv.	seed
*C*	0.245	*9.1*	0.004	*0.911*	0.014	0.019
LWP	17.4 g m^−2^	5.5	0.069	0.078	0.104	0.136
RWP	0.72 g m^−2^	*85.8*	0.067	0.077	0.033	0.199
*z*_*b*_	1 km	5.5	0.010	*0.756*	0.025	0.082
*z*_*i*_	2.3 km	1.0	0.017	*0.784*	0.008	0.034
*P*_srf_	0.17 W m^−2^	*230.7*	0.160	0.006	0.008	0.135
*w*_*q*_	0.0538 g kg^−1^ m s^−1^	0.7	0.000	*0.966*	0.001	0.009
*w*_*θ*_	0.00585 K m s^−1^	1.3	0.026	*0.872*	0.002	0.041
*τ*	2.91 h	10.9	0.038	0.209	0.640	0.001

### (c) Poisson solver

**Figure 3.**
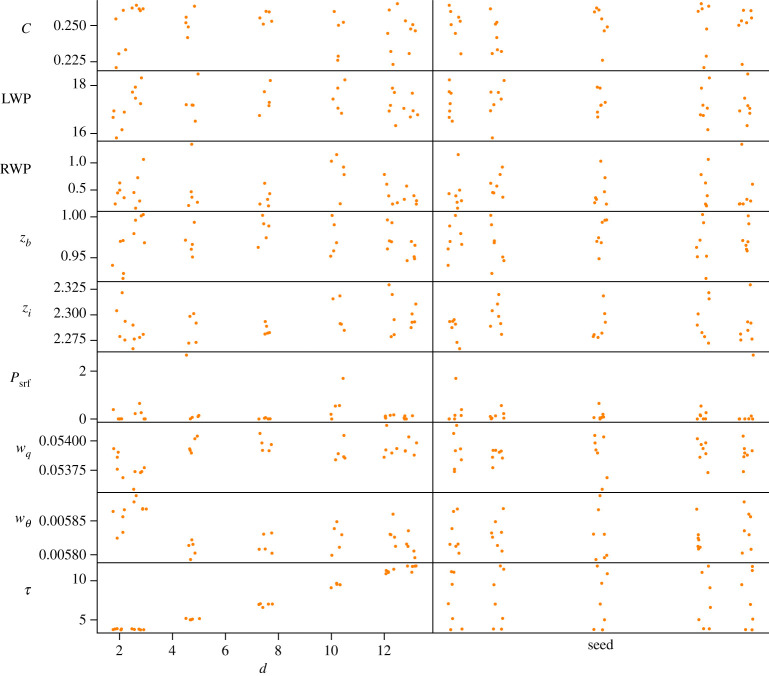
Influence of varying the numerical settings (horizontal) on model output quantities (vertical). The varied parameters are the Poisson solver tolerance *ϵ* = 10^−*d*^ and the random seed. (Online version in colour.)

The Poisson solver tolerance *d* is varied in figure 3 and table 4. The standard deviation of the surface precipitation *P*_srf_ and the rain water path RWP and the computational time are the largest. Of these, the computational time strongly depends on the Poisson-solver tolerance as seen from the Sobol indices, while the other two depend on both the random seed and the tolerance. In the plots in figure 3, the tolerance can be seen to affect the QoIs when d≲5, for larger *d* (smaller tolerances) the influence of *d* is hard to distinguish from the effects of the random seed. We conclude that a precision of about five digits seems sufficient in this case, resulting in a substantially shorter running time than for 13 digits—the highest precision tested (which is close to the limits imposed by double-precision floating point numbers). For the PALM LES model, a precision of four digits is reported to be sufficient in general [34].

**Table 4.** Statistics and Sobol indices for varying the stopping tolerance *ϵ* = 10^−*d*^ of the iterative Poisson solver and the random seed. The italics quantities are discussed in the text.

QoI	mean	s.d. (%)	*d*	seed
*C*	0.25	5.1	0.490	0.037
LWP	17.4 g m^−2^	2.7	0.296	0.084
RWP	0.551 g m^−2^	*54.0*	0.567	0.043
*z*_*b*_	0.978 km	1.8	0.230	0.079
*z*_*i*_	2.29 km	0.7	0.414	0.037
*P*_srf_	0.205 W m^−2^	*157.6*	0.254	0.083
*w*_*q*_	0.0539 g kg^−1^ m s^−1^	0.2	0.662	0.034
*w*_*θ*_	0.00583 K m s^−1^	0.4	0.694	0.053
*τ*	7.24 h	*34.3*	*0.995*	0.002

## 4. Conclusion

From the UQ analysis of the RICO simulation with the DALES model, we can conclude which parameters and model settings are associated with the largest uncertainty in the model outputs. Of the physical parameters varied, the sea surface temperature is the most influential. The rain quantities shows a strong dependency on the cloud droplet concentration, and also large variations between runs started with different random initial states. Of the model choices explored, the choice of advection scheme was the most influential, and had particularly strong effects on the cloud cover.

In the RICO study [11] comparing several models, RWP and surface precipitation were seen to vary strongly from model to model, while the other model quantities agree much more closely. Our analysis of DALES shows that these same quantities vary strongly from one model run to the next, i.e. when initialized with different random seeds. The variation in RWP and precipitation seen in the RICO study may thus be a result of variability in these quantities, rather than robust differences between the models. This shows the importance of running multiple simulations with different random seeds, to quantify the uncertainties from the random initial state, which of course comes with a significant increase in the computational cost. Seifert & Heus [39] note that the simulation domain in [11] is so small that it can support only one significant rain event at once, leading to large variability of precipitation over time. To mitigate this, they run the same case with both larger domains and higher resolutions. Larger domains lead to some self-averaging, and can be expected to produce less variability from one run to the next. On the other hand, larger domains permit organization over larger scales (and there are hints that LES simulations organize into larger and larger structures if run unperturbed for long enough) [9]. Thus increasing the domain size is not guaranteed to eliminate fluctuations between runs started from different initial states.

When comparing models to each other, or when measuring dependencies on parameter values, the spread of the results should be compared with the uncertainty arising from the model randomness. A minimal practical advice for future studies, when a full UQ study is not computationally feasible, would be to repeat at least some of the simulations with identical settings except for different random initial states, to have at least an estimate of the variability in the different QoIs.

For a more detailed view of the model uncertainties, one could consider vertical profiles as the QoIs for analysis, to show where in the atmosphere the parameters are influential. Further experiments could benefit from more advanced UQ methods, to reduce the computational burden that comes with a higher number of uncertain coefficients. For instance, sparse-grid methods construct a sampling plan by taking a linear combination of tensor products of 1D collocation points of increasing order. In high dimensions, this can lead to a much more efficient sampling plan compared to the current approach of taking a single tensor product of 1D collocation points of a given fixed order. That said, sparse grids are still isotropic, in the sense that they treat each input dimension the same, and the results of this article clearly indicate that some inputs are more important than others. This shortcoming can be alleviated by considering dimension-adaptive approaches, which build a sampling plan in several iterations. At each iteration a suitable error metric is computed, based on which it is decided which input dimension needs refinement, e.g. [40]. Both these approaches are currently being implemented in EasyVVUQ, and will be available for future research. A further form of model input that would be interesting to vary in a UQ experiment is the initial vertical profiles of the model state quantities.

## Data accessibility

EasyVVUQ, DALES, FabSim3 and the EasyVVUQ-DALES script are available under open-source licenses: DALES https://github.com/dalesteam/dales, doi:10.5281/zenodo.3759192; EasyVVUQ https://github.com/UCL-CCS/EasyVVUQ, DOI: 10.5281/zenodo.3722091; FabSim3 https://github.com/djgroen/FabSim3; EasyVVUQ-DALES script https://github.com/fjansson/EasyVVUQ-DALES.
